# Decannulation: a retrospective cohort study of clinical and swallowing indicators of success

**DOI:** 10.1016/j.clinsp.2022.100071

**Published:** 2022-06-24

**Authors:** Carina Escudero, Fernanda Chiarion Sassi, Gisele Chagas de Medeiros, Maíra Santilli de Lima, Paulo Francisco Guerreiro Cardoso, Claudia Regina Furquim de Andrade

**Affiliations:** a Departamento de Fisioterapia, Fonoaudiologia e Terapia Ocupacional da Faculdade de Medicina da Universidade de São Paulo, São Paulo, SP, Brazil; b Divisão de Fonoaudiologia do Instituto Central do Hospital das Clínicas da Faculdade de Medicina da Universidade de São Paulo, São Paulo, SP, Brazil; c Departamento Cardiopneumologia, Disciplina de Cirurgia Torácica da Faculdade de Medicina da Universidade de São Paulo, São Paulo, SP, Brazil

**Keywords:** Swallowing, Swallowing disorders, Tracheostomy, Decannulation, Speech therapy

## Abstract

**Objective:**

To investigate the clinical and swallowing indicators related to a successful decannulation process during the hospital stay.

**Methods:**

A retrospective cohort clinical study. The study sample comprised a heterogeneous patient population who had submitted to a tracheostomy procedure in a tertiary hospital. Patients were divided into two groups (decannulated vs. non-decannulated) and compared not only in terms of demographic and clinical data but also the results of a swallowing assessment and intervention outcome.

**Results:**

Sixty-four patients were included in the present study: 25 (39%) who had been successfully decannulated, and 39 (61%) who could not be decannulated. Between-group comparisons indicated that both groups presented similar clinical and demographic characteristics. The groups also presented similar swallowing assessment results prior to intervention. However, significant differences were observed regarding the time to begin swallowing rehabilitation. The decannulated group was assessed nine days earlier than the non-decannulated group. Other significant differences included the removal of the alternate feeding method (72.0% of decannulated patients vs. 5.1% of non-decannulated patients) and the reintroduction of oral feeding (96.0% of decannulated patients vs. 41.0% of non-decannulated patients) and functional swallowing level at patient disclosure. The non-decannulated patient group presented higher death rates at disclosure.

**Conclusion:**

The results of the present study indicated that the following parameters were associated with a successful decannulation process: early swallowing assessment, swallowing rehabilitation, and improvement in the swallowing functional level during the hospital stay. The maintenance of low swallowing functional levels was found to be negatively associated with successful decannulation.

## Highlights

• Deccanulation indicators were investigated in patients who were submitted to a tracheostomy procedure.
• Early swallowing evaluation and rehabilitation were associated with a successful decannulation process.
• Low swallowing functional levels were negatively associated with the success of decannulation.

## Introduction

According to the literature, approximately 10% to 15% of the individuals admitted to Intensive Care Units (ICU) and under prolonged mechanical ventilation may require the use of tracheostomy.^1^, ^2^, ^3^, ^4^ The advantage of tracheostomy over OTI is improved overall patient comfort and less need for intensive care.^2^ Despite the benefits, tracheostomy may lead to immediate and late complications, such as bleeding, stoma infection, pneumothorax, dysphagia, laryngotracheal stenosis, and tracheomalacia, tracheoesophageal fistula, pneumonia, and aspiration.^1^^,^^5^^,^^6^

As for dysphagia, there is no consensus in the literature about the effects of the tracheostomy tube on swallowing.^7^ Recent studies have pointed out that, in tracheostomized patients, dysphagia is usually associated with the underlying disease and not with the presence of the tracheostomy tube itself.^8^ However, a few authors have suggested that the presence of the tracheostomy tube is associated with the reduction in the hyolaryngeal excursion, subglottic pressure, airway protective reflexes, and a reduction in the laryngeal and pharyngeal sensitivity. Besides that, the atrophy of the laryngeal muscles caused by the presence of the tube and the external pressure of the cuff exerted on the esophagus, has been related to changes in the pharyngeal phase of swallowing.^9^

Regarding the tracheostomy removal process, although the literature contains no well-established guidelines, there is some consensus therein regarding related parameters, and they should be considered.^10^ The first of these parameters is whether there is a complete resolution of the issue that triggered the need for tracheostomy in the first place, along with whether it is necessary to continue using MV, including for procedures that require the use of anesthesia.^1^^,^^6^ It is essential that clinical assessments of swallowing and respiratory function be performed by checking the ability to manage saliva, secretions, and airway patency.^10^^,^^11^ Additionally, the patient must be able to tolerate the occlusion of the tracheostomy tube, with the cuff deflated, for more than 24 hours.^1^^,^^10^^,^^12^ If there is a positive result for all these conditions and the patient shows a satisfactory state of consciousness and an effective cough, decannulation is recommended.^1^^,^^10^^,^^12^ Chest X-Ray exams, fiber-optic bronchoscopy, nasolaryngofibroscopy, tomography, videofluoroscopy of swallowing, and swallowing videoendoscopy can also assist in making this decision.^11^^,^^13^ The presence of moderate to severe dysphagia and silent aspiration are considered factors of decannulation failure,^13^^,^^14^ as well as difficulties with expectoration/increased secretion, presence of tracheal stenosis and pulmonary infection,^13^^,^^15^ and advanced age (over 60 years).^12^, ^13^, ^14^^,^^16^, ^17^, ^18^^,^^20^

The decision to remove the tracheostomy tube is a multiprofessional process. Considering that there is a gap in the literature regarding prognostic markers related to swallowing and to the role of a swallowing rehabilitation process in the decannulation process, the present study aimed to investigate the clinical and oral motor indicators related to the success of decannulation in patients during the hospital stay.

## Material and methods

This is a retrospective cohort clinical study approved by the Ethics Committee for Analysis of Research Projects of the Institution (CAPPesq – Process nº 3.687.677).

### Participants

This study examined patients hospitalized at the Centra*l* Institute of Hospital das Clínicas, School of Medicine of the University of São Paulo, Brazil. (ICHC FMUSP) between January 2019 and March 2020; all had undergone tracheostomy and were referred to the Division of Oral Myology for assessment and rehabilitation.

The inclusion criteria adopted were: a) Use of tracheostomy, b) Age ≥18 years; c) Glasgow Coma scale score ≥13, d) Orotracheal intubation and tracheostomy performed during the hospital stay, e) Independence of continuous invasive MV. The adopted exclusion criteria were: a) Previous surgical procedures involving the head and neck region, b) Subglottic stenosis prior to hospitalization, c) Neurodegenerative disease, d) New surgical procedures requiring the use of general anesthesia; and e) Missing data in the medical records.

Considering decannulation was the primary outcome of this study, the data obtained were divided into two groups, for the analysis of decannulation success parameters: decannulated vs. non-decannulated participants. At ICHC-FMUSP, the decision of when to begin tracheostomy weaning has a multidisciplinary approach, and predictors of failure must be absent. First of all, the primary factor that led to the indication of a tracheostomy must be resolved. Moreover, the patient must not be ventilator-dependent; should present ≥8 points on the Glasgow Coma Scale and absence of delirium; should present hemodynamic and ventilatory stability; should demonstrate no signs of active infection; and should not be scheduled for new surgical interventions involving the use of general anesthesia. If all of these criteria are present, the patient will be referred to a swallowing assessment in order to verify the swallowing function (i.e., clinical assessment of the oral motor structures, swallowing biodynamics) and the possibility of maintaining a deflated cuff. For this step, the Blue Dye Test is used. It is also necessary for the physical therapy team to evaluate the presence of the spontaneous cough reflex, the patient's ability to clear secretions, and the effectiveness of coughing itself. These aspects are evaluated through the peak expiratory flow meter test (i.e., reference value ≥ 40 cm H_2_O). If the patient passes all of these criteria and there is an indication to proceed with decannulation, the physician will evaluate the presence of any obstructive lesions in the larynx and trachea, and will perform the airway patency test and the intra-airway pressure measurement. The greater the patient's compliance to the above criteria, the greater the chances of having a successful decannulation. It is important to highlight, that in the present study patients included in the non-decannulated group did not achieve the necessary conditions, and, therefore, no attempts of decannulation were made.

### Procedures

#### Clinical indicators

The demographic and clinical variables included in the study were age, gender, underlying disease, classification of the patient's severity level according to the Simplified Acute Physiology Score (SAPS-III) scale,^21^ number of OTIs, translaryngeal intubation duration, MV duration (in days), days between the placement of the tracheostomy tube and the initial oral motor assessment, days between MV weaning and initial oral motor assessment, the primary outcome (decannulation status: decannulated vs. non-decannulated), and secondary outcome (swallowing therapy discharge, hospital discharge, suspension of swallowing therapy due to worsening of the clinical condition, hospital transfer, or death).

As for the swallowing variables, the following clinical data and indicators were included: clinical assessment of swallowing, with the determination of the functional level of swallowing according to the American Speech-Language-Hearing Association National Outcome Measurement System (ASHA NOMS)^22^ at two different moments ‒ at the initial oral motor assessment and at the outcome; type of feeding method at initial assessment and at the outcome (oral feeding, nasoenteral tube, or gastrostomy); duration of alternative feeding method until the oral motor assessment; the number of swallowing rehabilitation sessions for removal of the alternate feeding method and reintroduction of oral feeding; the number of swallowing rehabilitation sessions until the outcome.

#### Functional swallowing level

The authors used the ASHA NOMS scale to determine the functional level of swallowing.^22^ The ASHA NOMS swallowing level scale is a multidimensional tool designed to measure both the supervision level required and diet level by assigning a single number between 1 to 7, with the lowest score indicating greater impairment in swallowing (i.e., level 1 ‒ Inability to safely swallow by mouth. All nutrition and hydration are received via an alternative feeding method; level 7 ‒ the individual's ability to eat independently is not limited by the swallowing function. Swallowing is safe and efficient for all consistencies. Compensatory strategies are executed effectively when needed). In this study, the authors considered the ASHA NOMS classification at the time of the initial oral motor assessment and for each patient's secondary outcome.

#### Speech therapy intervention

All patients received individual treatment for swallowing rehabilitation until the resolution of dysphagia and decannulation, or until the final outcome. Patients were treated by an experienced SLP who had been trained to apply for the same treatment program. Treatment (i.e., direct, and indirect therapies) leveraged procedures and techniques aimed at swallowing rehabilitation. Direct therapy is based on the use of food, even in minimal volumes, to provide swallowing training, while indirect therapy focuses on muscle coordination and uses exercises for oral motor training.

### Data analysis

The data collected was submitted to statistical analysis using SPSS software version 27. Quantitative data underwent descriptive analysis (medians and percentiles) and inferential analysis that compared the groups (Mann-Whitney *U* test). Qualitative data underwent descriptive analysis (total count and percentage) and inferential analysis that compared the two groups (Pearson's Chi-Square test). The significance level adopted in all analyses was 5%.

The estimation of the survival distribution was performed using the Kaplan-Meier log-rank test. The successful decannulation after swallowing rehabilitation was defined as the outcome, and the exposure groups were defined considering the time interval between MV independence and swallowing assessment. Censored observations were the non-decannulated individuals.

## Results

During the study period, 106 tracheostomized patients were evaluated by the Division of Oral Myology, out of which 64 (25 decannulated individuals during the hospital stay and 39 non-decannulated individuals) met the inclusion and exclusion criteria established for this study. Considering the underlying disease, the decannulated group had the following diagnoses: neurological diseases (n = 19), burns (n = 3), liver transplantation (n = 2), and cervical abscess (n = 1). For the non-decannulated group, the diagnoses were: neurological diseases (n = 32), lung diseases (n = 2), cervical abscess (n = 2), cutaneous focus septic shock (n = 2), and burns (n = 1).

**Fig. 1.**
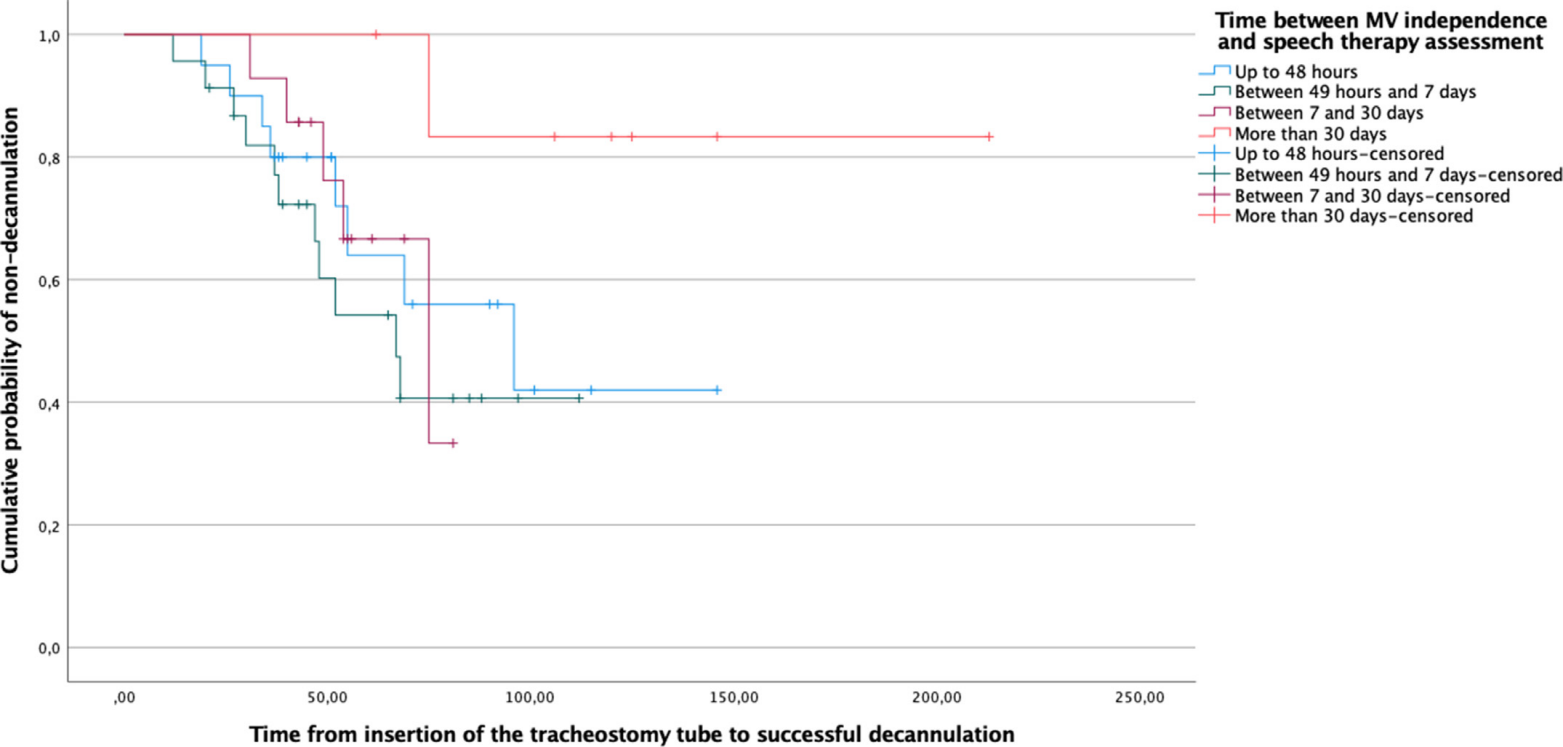
Comparation of Kaplan Meier probability curves for time from insertion of the tracheostomy tube to successful decannulation, by the time between MV independence and swallowing assessment.

Table 1 presents the clinical and demographic data of the studied population. With the exception of the parameter time between MV weaning and initial oral motor assessment, the variables were similar between groups. The results of the Kaplan-Meier log-rank test presented in Fig. 1 confirmed that the delay between MV independence and swallowing assessment significantly affected the time to successful decannulation.

**Table 1.** Intergroup comparison of demographic variables and clinical data.

	Decannulated individuals (n = 25)	Non-decannulated individuals (n = 39)	p-value
**Age (years)**			
median (P25; P75)	43.0 (35.5; 61.0)	52.0 (43.0; 61.0)	0.24
**Sex, n (%)**			
Male	15 (60.0%)	28 (71.8%)	0.69
Female	10 (40.0%)	11 (28.2%)	
**SAPS-3 score**			
median (P25; P75)	53.0 (44.0; 61.0)	52.0 (46.0; 65.0)	0.69
**Length of hospital stay (days)**			
median (P25; P75)	75.0 (55.0; 97.5)	65.0 (45.0; 97.0)	0.51
**Number of OTIs**			
median (P25; P75)	1.0 (1.0; 1.0)	1.0 (1.0; 2.0)	0.44
**Translaryngeal intubation duration (days)**			
median (P25; P75)	13.0 (8.5; 14.0)	13.0 (9.0; 18.0)	0.59
**Time between placement of TCT and speech therapy assessment (days)**			
median (P25; P75)	6.0 (3.0; 14.0)	13.0 (4.0; 27.0)	0.27
**MV duration time (days)**			
median (P25; P75)	15.0 (13.0; 25.0)	16.0 (10.0; 22.0)	0.76
**Time between MV independence and speech therapy assessment (days)**			
median (P25; P75)	4.0 (2.0; 7.5)	5.0 (2.0; 23.0)	0.042^a^
**Time from first SLT assessment to successful decannulation (days)**			
median (P25; P75)	47.0 (30.5; 61.0)	‒	‒

n, number of participants; P25, 25^th^ percentile; P75, 75^th^ percentile; SAPS-3, Simplified Acute Physiology Score – third version; OTI, Orotracheal Intubation; TCT, Tracheostomy; MV, Mechanical Ventilation.

a Significant difference according to the Mann-Whitney *U* test. ** Significant difference according to Pearson's Chi-Square test.

Table 2 describes the results for the swallowing variables. Significant differences were observed between the groups in terms of the number of speech therapy sessions until the patient's outcome and for data related to the use and withdrawal of the alternative feeding method and the reintroduction of oral feeding. Furthermore, significant differences were observed for almost all outcome variables. It is important to observe that the non-decannulated group had a higher number of deaths and a higher rate of swallowing therapy suspension due to the worsening of the case.

**Table 2.** Intergroup comparison for the swallowing and outcome indicators.

	Decannulated individuals (n = 25)	Non-decannulated individuals (n = 39)	p-value
**Total number of swallowing rehabilitation sessions until the outcome**			
median (P25; P75)	13.0 (10.0; 22.5)	8.0 (3.0; 15.0)	0.005^a^
**Alternative feeding method in the initial speech therapy assessment, n (%)**			
Fasting	0 (0.0%)	1 (2.6%)	0.68
NES	23 (92.0%)	34 (87.2%)	
GTT	2 (8.0%)	4 (10.3%)	
**Time of use of alternative feeding method in the initial swallowing assessment (days)**			
median (P25; P75)	18.0 (11.0; 31.0)	21.0 (11.0; 35.0)	0.62
**Alternative feeding method in the outcome, n (%)**			
GTT	1 (4.0%)	14 (35.9%)	<0.001^b^
OF	18 (72.0%)	4 (10.3%)	
GTT + OF	4 (16.0%)	5 (12.8%)	
NES	0 (0.0%)	11 (28.2%)	
NES + OF	2 (8.0%)	5 (12.8%)	
**Number of participants who underwent reintroduction oral feeding during swallowing rehabilitation, n (%)**	24 (96.0%)	16 (41.0%)	<0.001^b^
**Number of swallowing rehabilitation sessions until return to oral feeding**			
Median (P25; P75)	4.0 (2.5; 9.0)	5.0 (2.0; 13.0)	0.81
**Number of participants who removed alternative feeding method during swallowing rehabilitation, n (%)**	18 (72.0%)	2 (5.1%)	<0.001^b^
**Outcome, n (%)**			
Swallowing therapy discharge	13 (52.0%)	10 (25.6%)	0.032^b^
Hospital discharge	9 (36.0%)	11 (28.2%)	0.51
Service suspension	0 (0.0%)	9 (23.1%)	0.010^b^
Transfer to another hospital	3 (12.0%)	11 (28.2%)	0.12
Death	0 (0.0%)	7 (17.9%)	0.025^b^

n, Number of participants; P25, 25^th^ percentile; P75, 75^th^ percentile; NES, Nasoenteral tube; GTT, Percutaneous Endoscopic Gastrostomy Tube; OF, Oral Feeding.

a Significant difference according to the Mann-Whitney *U* test.

b Significant difference according to Pearson's Chi-Square test.

Tables 3 and 4 show the variation in swallowing and distribution of patients among the ASHA NOMS levels in the initial assessment and outcome. The results indicated that the groups did not differ significantly during the initial swallowing assessment. Although both groups presented a significant improvement in the functional level of swallowing (i.e., p < 0.05 according to the Wilcoxon signed-rank test, comparing the results on the initial assessment to the outcome), this improvement was greater in the decannulated group (i.e., safe swallowing with minimal dietary restrictions) when compared to the non-decannulated group (i.e., need for an alternative feeding method).

**Table 3.** Intergroup comparison regarding swallowing functional level, at the initial swallowing assessment and at the outcome.

	Decannulated individuals (n = 25)	Non-decannulated individuals (n = 39)	p-value
**ASHA NOMS total score – initial assessment**			
Median (P25; P75)	2.0 (1.5; 3.0)	2.0 (1.0; 2.0)	0.25
**ASHA NOMS total score – outcome assessment**			
Median (P25; P75)	7.0 (4.5; 7.0)	2.0 (1.0; 4.0)	<0.001^a^

P25, 25^th^ Percentile; P75, 75^th^ Percentile.

a Significant difference according to the Mann-Whitney *U* test.

**Table 4:** Distribution of participants among the swallowing functional levels, at the initial swallowing assessment and at the outcome.

ASHA NOMS level	Initial assessment		Outcome assessment	
	Decannulated individuals (n = 25)	Non-decannulated individuals (n = 39)	Decannulated individuals (n = 25)	Non-decannulated individuals (n = 39)
1	6 (24.0%)	12 (30.7%)	0 (0.0%)	10 (25.6%)
2	12 (48.0%)	21 (53.8%)	1 (4.0%)	14 (35.8%)
3	4 (16.0%)	5 (12.8%)	1 (4.0%)	5 (12.8%)
4	1 (4.0%)	1 (2.5%)	4 (16.0%)	5 (12.8%)
5	1 (4.0%)	0 (0.0%)	3 (12.0%)	4 (10.2%)
6	0 (0.0%)	0 (0.0%)	3 (12.0%)	1 (2.5%)
7	1 (4.0%)	0 (0.0%)	13 (52.0%)	0 (0.0%)

## Discussion

In general, the results of the present study indicated that early swallowing intervention (i.e., soon after weaning from MV, if the patient maintains clinically stable), swallowing rehabilitation, safe return to oral feeding (i.e., number of speech therapy sessions), and improvement of the swallowing functional level are determining factors for the success of the decannulation process.

In the present study, 39% of all patients were decannulated during their hospital stay. This result is lower than other studies that showed decannulation rates ranging from 45% to 72%.^2^^,^^17^^,^^19^^,^^21^, ^22^, ^23^^,^^26^ A possible explanation for this difference may lie in the reason for hospitalization: in this study, the neurological disease was generally the main reason for tracheostomy. According to the literature, patients with neurological diseases (e.g., stroke and traumatic brain injury) have a lower decannulation success rate.^11^^,^^12^^,^^19^^,^^21^^,^^22^

Regarding the clinical and demographic factors, neither the translaryngeal intubation duration nor the MV duration differed between the studied groups of patients. This suggests that the time to perform tracheostomy and MV does not seem to change the prognosis of a successful decannulation. This result corroborates the literature, which does not present a consensus on the best time to perform the tracheostomy.^1^ Blot et al.^23^ did not find a significant difference among the rates of acquired pneumonia, mortality, length of ICU stay, and time without MV when comparing patients who underwent early and late decannulation. However, differences between these groups were observed regarding comfort, since patients who performed an early tracheostomy (i.e., removed the orotracheal tube earlier) were able to start the rehabilitation process sooner, thus accelerating bed to chair transfer, oral feeding reintroduction, and improving overall communication. Moreover, it should be observed that the literature proposes that neurological patients ‒ who comprise the majority of the sample ‒ can benefit from early tracheostomy (i.e., 7–8 days after admission).^1^ In the present study, the average time to perform a tracheostomy was 13 days, which is therefore considered a late tracheostomy.^1^ Still, this average time is similar to the world average (14 days) as per data from 459 ICUs in 50 countries.^4^

Additionally, in relation to the clinical parameters, significant differences were observed between groups in terms of MV independence time and referral for a swallowing assessment. The decannulated group, on average, was submitted nine days sooner to a swallowing assessment than the non-decannulated group. This indicates that undertaking swallowing rehabilitation earlier increases the likelihood of tracheostomy removal. Zanata et al.^12^ demonstrated that swallowing rehabilitation in tracheostomized patients after traumatic brain injury reduces both the time of tracheostomy use and the length of hospital stay. Other studies have also demonstrated that a multidisciplinary approach to patients in the use of a tracheostomy tube, which includes a swallowing rehabilitation program, increases the chances of decannulation,^24^ decreases the time for the removal of the tracheostomy tube,^25^ improves the quality of life as the patient is able to communicate better and reduces the time of ICU stay.^26^ Furthermore, implementing an early swallowing intervention program for patients at risk for bronchopulmonary aspiration reduces the risk of acquired pneumonia and improves patients’ overall health condition.^27^ Therefore, the group of decannulated patients included in the present study may have experienced fewer pulmonary complications in comparison to the non-decannulated group, justifying the more favorable outcome.

Regarding the clinical indicators, the present results indicated that during the first swallowing assessment, 93.7% of the tracheostomized population had severe dysphagia (i.e., low swallowing functional levels – ASHA NOMS levels 1–3). These findings are similar to those of Hakiki et al.,^20^ who found a 92.4% rate of severe dysphagia in patients with an acquired brain injury and who were using tracheostomy. The high rate of patients with severe dysphagia in this study can be explained not only by the presence of neurological disease but also by the prolonged translaryngeal intubation duration,^26^^,^^28^ considering that the mean translaryngeal intubation duration was 12.2 days for decannulated patients and 14 days for non-decannulated patients. Explanations for the association between prolonged translaryngeal intubation duration and dysphagia mainly relate to the length of time the tube remains in the oral cavity, pharynx, and larynx: a longer time period reduces the response of airway protective reflexes and possibly causes damage to the mechanoreceptors responsible for triggering the swallowing reflex.^28^

Although the groups did not differ in terms of the swallowing functional level during the initial swallowing assessment, significant differences were observed between the groups at the time of the outcome. The authors observed that 96% of the decannulated patients were reintroduced to oral feeding, and 72% had the alternative feeding method removed. On the other hand, 64% of the patients in the non-decannulated group maintained the exclusive use of an alternative feeding method. These data suggest that the improvement of the functional level of swallowing during swallowing rehabilitation is a determining factor for decannulation. Other studies highlight the relationship between decannulation success and the ability to swallow saliva, food, and liquids.^8^^,^^17^, ^18^, ^19^^,^^29^ In addition, oral feeding introduction strongly correlates with decannulation time.^8^

Besides presenting more favorable swallowing rehabilitation outcomes, individuals who have the tracheostomy removed are more likely to experience hospital and speech therapy discharge, while those not decannulated had higher death rates. According to the literature, the probability of a patient's decannulation closely relates to their ability to manage respiratory secretions properly.^8^ Additionally, the literature also points to an increased risk of morbidity and mortality among patients who leave the ICU with a tracheostomy tube intact.^8^ One of the factors related to the increased mortality of hospitalized tracheostomized patients is a high body-mass index; however, data on this parameter were not recoverable in this study.

Finally, this study did have some limitations. The sample of participants included in the study was heterogeneous and derived from a single institution; therefore, the results herein may reflect only the characteristics of the procedures adopted at this location. Moreover, the low sample size prevented the performance of a multivariable analysis adjusting for known illness severity factors. In addition, the competing risk of death or being transferred to long-term hospitals among the non-decannulated patients could be considered a possible bias, since the more severe patients ‒ not detected by the initial SAPS-3 score ‒ are less likely to be decannulated due to inherent patient characteristics. However, one should consider that this group of patients reflects the reality of an oral myology service within a high-complexity tertiary hospital. Another limiting factor was the retrospective nature of the study, which allowed the retrieval only of data recorded in medical records. It is suggested that future studies should prospectively follow up tracheostomized patients, investigating clinical factors such as body mass index, amount, and capacity to handle secretions, cough strength, in-hospital clinical complications, and post-discharge follow-up.

## Conclusion

Considering that the analysis performed in the present study was bivariate and did not account for other variables such as the impact of underlying health conditions, the results of the present study indicated the that the following parameters were associated with successful decannulation process: early swallowing assessment, swallowing rehabilitation, and improvement in the swallowing functional level during the hospital stay. The maintenance of low swallowing functional levels was found to be negatively associated with successful decannulation. The findings of the present study are exploratory because confounding and the competing risk of death could not be accounted for.

## Authors’ contributions

Carina Escudero: Responsible for data collection and analysis, and interpretation of the results.

Fernanda Chiarion Sassi: Responsible for supervising the research; organizing the statistical analyses; interpretation of the results; writing a major portion of the paper.

Gisele Chagas de Medeiros: Contributed to data analysis and interpretation; contributed to manuscript preparation.

Maíra Santilli de Lima: Responsible for data collection and analysis, and interpretation of the results.

Paulo Francisco Guerreiro Cardoso: Contributed to data analysis and interpretation; responsible for revising the final version of the manuscript.

Claudia Regina Furquim de Andrade: Responsible for the research and experimental design; contributed to data analysis and manuscript preparation.

## Financial support

None.

## Conflicts of interest

The authors declare no conflicts of interest.
